# Hemorrhagic Lacrimation and Epistaxis in Acute Hemorrhagic Edema of Infancy

**DOI:** 10.1155/2016/9762185

**Published:** 2016-11-14

**Authors:** Shireen Mreish, Hossam Al-Tatari

**Affiliations:** Pediatric Department, Tawam Hospital, Al Ain, UAE

## Abstract

Acute hemorrhagic edema of infancy is an uncommon benign cutaneous vasculitis. Despite its worrisome presentation, it carries good prognosis with rarely reported systemic involvement. Management of these cases has been an area of debate with majority of physicians adopting conservative modalities. We report a case that presented with classic triad of rash, low grade fever, and peripheral edema along with two rarely reported manifestations in literature: hemorrhagic lacrimation and epistaxis.

## 1. Introduction

Acute hemorrhagic edema of infancy is an uncommon benign cutaneous vasculitis of the dermal small vessels [1]. The classic presentation is a triad of low grade fever and ecchymotic rosette like rash that tends to rapidly progress starting on the ears then to the face and limbs, with nonpitting and occasionally indurating tender acral edema [1]. Despite its worrisome presentation, the condition is self-limited and the course of the illness is benign without visceral involvement in most cases. Hence most experts recommend no treatment to be offered [1, 2]. We report a case presented to us with the classic triad along with hemorrhagic lacrimation and epistaxis, both of which are rarely reported manifestations.

## 2. Case Presentation

A 6-month-old previously healthy female infant, product of full term pregnancy and normal vaginal delivery, presented on her second day of illness with history of low grade fever and sudden onset of nonitchy red-purplish rosette like ecchymotic lesions on the face that progressed over a period of 2 days to the lower limbs involving soles, buttocks, and genital area. She also had history of associated mild nasal congestion. The patient was seen on the first day of illness in a pediatric clinic and was prescribed an oral antibiotic (amoxicillin). On the second day she had one episode of mild epistaxis and family reported bloody tears. Two days prior to appearance of the symptoms child received her scheduled 6-month vaccination (DTaP, Hib B, and Hepatitis B). There is no family history of similar cases or history of bleeding disorders. Review of her systems was negative except for what is mentioned in history of present illness.

**Figure 1:**
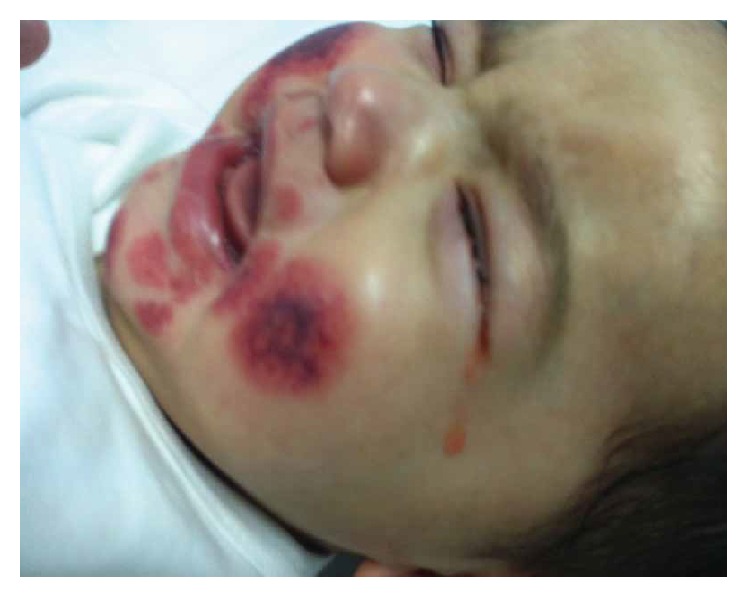
Bloody stained tears.

**Figure 2:**
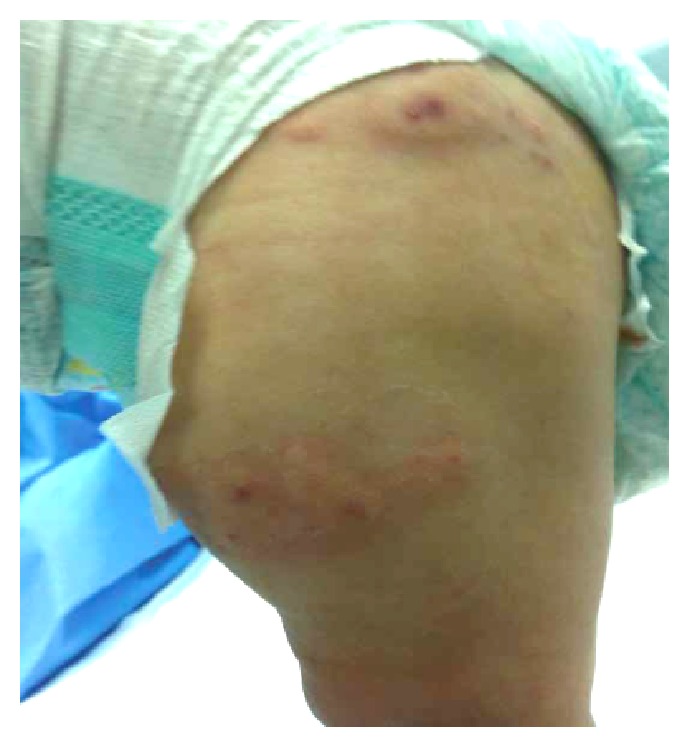
Swollen legs.

On presentation, her vital signs showed mild tachycardia 143/min and mild tachypnea 38/min (secondary to irritability) and normal blood pressure (90/50) and saturation. Her growth parameters were within normal range for her age. She was irritable and noticed to be crying with blood stained tears (Figure 1). Her face was puffy with edema of the ears, and her legs were noticed to be swollen too with no limitation of joint mobility (Figure 2). The rash was not tender edematous annular ecchymoses measuring from 0.5 to 4 centimeters in diameter, most of them were like target or rosette, and some were coalescent. Distribution was as described earlier including the soles. Rest of examination was unremarkable.

Blood investigations including complete blood count (Hb 110 g/L, WBC 16.8 × 10^9^/L, and platelets 430 × 10^9^/L) and clotting studies (PT 11.1 seconds, INR 0.90, and PTT 25.7 seconds), CRP (43 mg/L), liver and kidney function tests, urine analysis and culture, and blood culture were all normal. Skin biopsy findings were consistent with leukocytoclastic vasculitis. The walls of capillaries within the dermis were damaged with the presence of fibrin deposits. They were infiltrated by fragmented neutrophils with extravasation of red blood cells and eosinophils.

**Figure 3:**
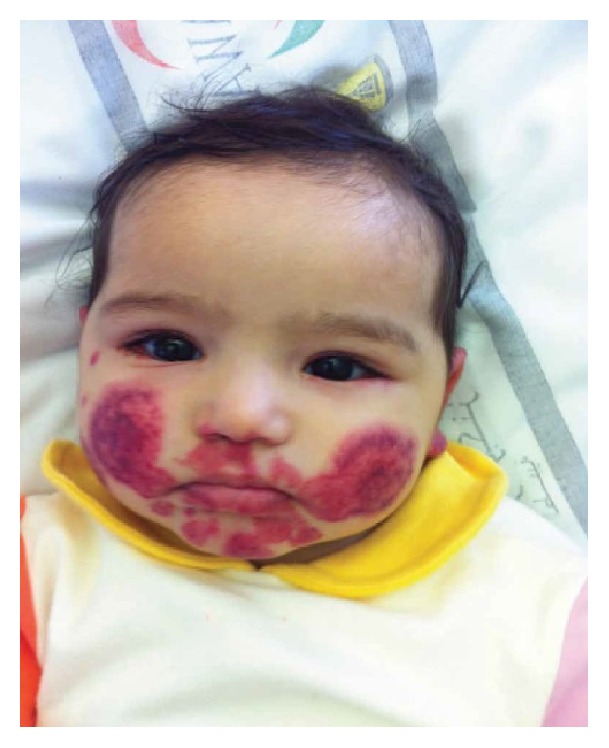
Day 2 after steroid treatment.

**Figure 4:**
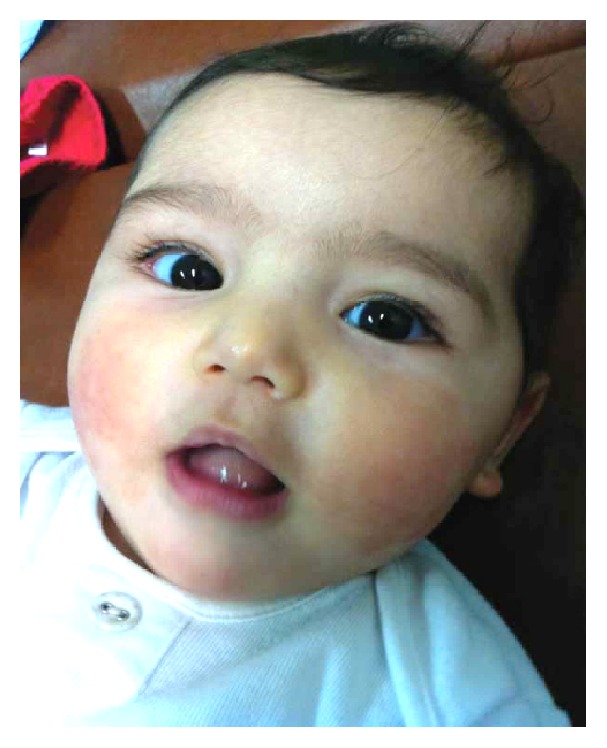
On follow-up day 15.

The patient received methylprednisolone IV (2 mg/kg/day) for 2 days, followed by prednisolone PO for 1 day (total 3 days of steroids) as well as oral antihistamine. She showed dramatic improvement in her condition after the first dose of steroids. She became less irritable, the swelling subsided, and rash progression was heralded (Figure 3). The patient was completely normal on follow-up two weeks later (Figure 4).

## 3. Discussion

AHEI was first described by Snow in 1913, and it was initially considered as a variant of HSP [3]. Nowadays it is considered by most authors as a separate entity for the infrequency of visceral involvement and immunoglobulin A (IgA) skin depositions, as well as the better prognosis compared to HSP [2]. There are no reported data on its incidence probably because of its rarity and confusion with other more common conditions, which might explain why it continues to be a challenging diagnosis for most pediatricians.

AHEI mostly affects children between the age of 2 and 24 months, with a male predominance of 2 : 1 ratio [1]. However, cases at birth and beyond 60 months of age were also reported [1, 4, 5]. The underlying pathophysiology is not well understood; however various causes have been identified as possible underlying etiology. Viral respiratory and gastrointestinal illnesses [1, 5, 6] are encountered in the majority of cases. Reported cases in literature identified chickenpox [7], herpes stomatitis [8], and pneumococcal infections [9] as infectious causes among other several pathogens. To a lesser extent, drug exposure especially antibiotics and scheduled vaccination may trigger the reaction [10–12]. The average interval between the onset of AHEI and the possible causative agent ranges from two days to one month [1].

Our patient had the classic triad at presentation. Her 6-month vaccination was most likely the trigger factor. She also developed hemorrhagic lacrimation and epistaxis, both of which are uncommonly reported manifestations. We found one reported case with similar manifestations after Rota virus infection [5]. Visceral and systemic involvement are uncommon in AHEI compared to HSP, yet serious complications have been reported such as gastrointestinal tract bleeding, lethal intestinal complications (e.g., intussusception) [13], renal involvement, auricular chondritis, epididymoorchitis [14], and unusual scarring [15].

Diagnosis of AHEI is made on clinical base. Hence, no investigations are necessary for diagnosis. However, being an uncommon condition, and because of the violent presentation of AHEI that may resemble other serious differential diagnoses like Kawasaki disease, erythema multiforme, HSP, and meningococcemia, basic laboratory investigations might be required. In most cases blood tests are normal as in our patient. However, leukocytosis, thrombocytosis, high CRP and ESR, and fibrinogenemia can be found occasionally [1, 4–7, 12].

Skin biopsy is only needed when diagnosis is uncertain. Histopathological findings of the biopsy may include perivascular infiltrates that are mainly composed of neutrophils with fragmented nuclei and occasionally eosinophils. There may also be endothelial edema with fibrin deposits in the vascular wall with a fibrinoid degeneration and erythrocyte extravasation [11, 15].

Treatment of AHEI has been always controversial, considering its benign and self-limited course. Spontaneous recovery usually occurs within 1–3 weeks [2], and up to 35 days in some cases [4]. Recurrences have been reported too [14].

We opted to treat with short course of steroids taking into consideration the presence of hemorrhagic lacrimation and epistaxis in our patient, both of which are unusual manifestation of AHEI. Despite the dramatic response that was noticed in patient's general condition and her lesions after the first dose, we are not certain if steroids treatment contributed to alleviating patient's symptoms. AHEI remains a benign cutaneous vasculitis where conservative management can be successful in the majority of cases.

## Abbreviations

AHEI: Acute hemorrhagic edema of infancy
HSP: Henoch-Schönlein purpura.
